# Molecular Detection and Characterization of *Giardia duodenalis* in Farmed Pigs in Three Provinces of Southern China

**DOI:** 10.3390/pathogens10111481

**Published:** 2021-11-14

**Authors:** Yang Zou, Xiao-Dan Yuan, Sheng-Ying Zhang, Hong-Yan Zhang, Xiao-Qing Chen

**Affiliations:** 1 Jiangxi Provincial Key Laboratory for Animal Health, College of Animal Science and Technology, Jiangxi Agricultural University, Nanchang 330045, China; zouyangdr@163.com; 2 State Key Laboratory of Veterinary Etiological Biology, Key Laboratory of Veterinary Parasitology of Gansu Province, Lanzhou Veterinary Research Institute, Chinese Academy of Agricultural Sciences, Lanzhou 730046, China; zhangsy1016@126.com (S.-Y.Z.); zhy7941@163.com (H.-Y.Z.); 3 College of Veterinary Medicine, Jilin University, Changchun 130062, China; yuanxiaodan0118@outlook.com

**Keywords:** *Giardia duodenalis*, prevalence, risk factors, farmed pigs, assemblages, southern China

## Abstract

*Giardia duodenalis* is a flagellated zoonotic parasite that can infect various animals and humans, causing economic losses in husbandry and detriments to public health. Although it has been reported in pigs worldwide, there are few reports on the prevalence and assemblages of *G. duodenalis* infection in pigs in China. In this study, the 396 pig fecal samples were randomly collected from seven farms in Zhejiang, Guangdong and Yunnan provinces in southern China, and were examined by means of the nested PCR amplification of β-giardin (bg), glutamate dehydrogenase (gdh), and triose phosphate isomerase (tpi) for the detection of *G. duodenalis*. Overall, 21 fecal samples were positive for *G. duodenalis*, with a prevalence of 5.3%. Three risk factors are associated with *G. duodenalis* infection, namely, region, age and gender. Moreover, 13, six and two samples were successfully amplified at the bg, gdh and tpi gene loci, respectively. Three assemblages of *G. duodenalis* were identified, including assemblage E (*n* = 17), assemblage A (*n* = 3) and assemblage B (*n* = 1). Assemblage E was the dominating genotype and was distributed in three provinces. These assemblages of *G. duodenalis* have also been found in human beings, non-human primates, sheep, goats and cattle, which further reveals that farmed pigs pose a potential threat to public health.

## 1. Introduction

*Giardia duodenalis* is a ubiquitous zoonotic intestinal parasite of animals and humans [1,2,3,4,5,6]. In humans, Giardiasis has been classified as a neglected parasitic disease by the WHO since 2004 [7]. *G. duodenalis* is known to cause infection in both humans and animals. While most of the infections are asymptomatic, infection in immunocompromised hosts causes diarrhea, vomiting and weight loss [8]. *G. duodenalis* is transmitted through the ingestion of food and water contaminated with cysts [9], or direct transmission. At the present, microscopy remains the method of choice for detecting *Giardia*; however, PCR analysis has a higher sensitivity [10,11].

*G. duodenalis* is classified into eight assemblages (A–H) based on molecular characterization [12]. Among them, assemblages A and B are commonly reported to infect humans [6,13,14], but also in animals, and can be transmitted zoonotically [12]. Other assemblages (C–H) have strong host specificities and narrow host ranges—assemblage C and assemblage D are usually detected in dogs and other canines. Assemblage E is identified in hoofed animals, and assemblage F and assemblage G are mostly detected in cats and rodents, respectively [12]. However, there are occasional exceptions to these host specificities. Assemblages such as assemblages C–F are reported in humans [15,16,17]. The animal-specific assemblages found in humans suggests that theses genotypes can also infect humans.

Organisms from infected farms such as *Escherichia coli*, *Rotavirus*, *Sapovirus*, coccidia, *Cryptosporidium parvum* and *G. duodenalis* [18,19] may contaminate the environment, soil and concrete surfaces and if attempts are not made to reduce or eliminate them, they may pose a threat to human health. Thus, the epidemiological investigation of these pathogens in pigs is important. Although some studies have reported *G. duodenalis* infection in pigs in the Xinjiang Uygur Autonomous Region, Henan Province, Shaanxi Province and Shanghai city, with prevalences ranging from 1.7% to 26.9% [19,20,21,22], there is limited information about *G. duodenalis* infection in pigs in southern China. Thus, the purpose of this study was to investigate the prevalence and assemblage distribution in pigs in Zhejiang, Guangdong and Yunnan provinces in southern China.

## 2. Results and Discussion

As the largest pork producer and consumer in the world, China has promoted the rapid development of a large-scale pig industry. Generally, the pigs infected with *G. duodenalis* infection may result in self-limited illness with weight loss and malabsorption, causing a decline in pig production. However, *G. duodenalis* is a relatively common parasite in pigs all over the world [19,20,21,22,23,24,25,26,27]. It can shed infectious cysts into the environment, which contaminate food and water, resulting in transmission to animals and humans [28,29]. Therefore, for the development of animal husbandry and the health of human beings, it is necessary to investigate the prevalence and assemblage distribution in pigs.

In this study, the total prevalence of *G. duodenalis* was 5.3% (95%CI: 3.09–7.51), which was lower than that of previous studies from Shaanxi Province, China (8.0%, 45/560) [19], Shanghai city (26.9%, 25/93) [22] and other countries (such as Poland, Nigeria, Denmark, Canada and the UK, where the prevalence ranged from 9.5 to 57.1%) [23,25,26,27,30], but higher than other studies from the Xinjiang Uygur Autonomous Region (2.6%, 21/802) [20], Henan Province (1.7%, 15/897) [21], Prince Edward Island, Canada (1%, 6/663) [26] and Norway (1.5%, 10/684) [31]. The various prevalences of *G. duodenalis* in pigs in these reports could be caused by regional differences, but differences in management methods, the number of samples and sampling seasons could also affect these results. Further epidemiological studies should be conducted to collect more data on the infection of pigs with *G. duodenalis* to analyze the causes of these differing prevalences. These findings provided fundamental information as to the epidemiological situation of *G. duodenalis* infection in pigs in southern China.

In addition, the prevalences compared between male (8.4%, 95% CI: 4.32–12.44) and female pigs (2.8%, 95% CI: 0.58–4.94) were significantly different (χ^2^ = 6.158, *df* = 1, *p* = 0.02). The significantly different prevalence found in gender groups of pigs (Table 1) is consistent with a previous study in Australia [23], but different from a study in the Shaanxi Province of China [19]. The reason for this difference is related to the different management system, animal stocking density, water supply and hygiene regimes [22]. Moreover, significantly different (χ^2^ = 9.942, *df* = 2, *p* = 0.01) prevalences were observed among different provinces, with the highest prevalence (10.5%, 95% CI: 5.09–15.87) observed in Zhejiang Province, and the lowest prevalence (2.5%, 95% CI: 0.34–4.66) observed in Yunnan Province, which was similar to previous reports [19,20]. A range of factors have been considered to influence the prevalence of *G. duodenalis* infection in pigs among different areas [19]. In addition, the prevalence of *G. duodenalis* infection in pigs aged 4–6 months (12.3%, 95% CI: 4.79–19.87) was significantly higher than that in pigs aged 1–3 months (2.3%, 95% CI: 0–5.45) (χ^2^ = 9.273, *df* = 2, *p* = 0.01) (Table 1). In fact, the immunity, gastrointestinal bacterial flora and nutritional status of the pigs were considered to be the causes of the varying age-related infection rates [32]. The present study indicated that there was significant difference among age groups (Table 1). This difference also has been found in pigs in Denmark [32]. 

**Figure 1:**
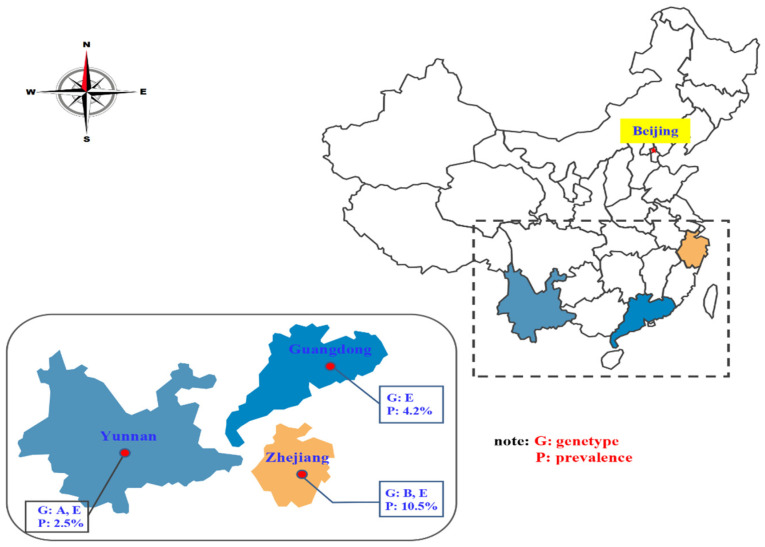
The distribution of assemblages and prevalence of *Giardia duodenalis* in pigs in three provinces.

To date, assemblages A, B (BIII, BIV), D and E have been reported in pigs worldwide [14]. In this study, 21 fecal samples were found to be *G. duodenalis*-positive by means of the PCR amplification of the SSU rRNA gene at the bg, gdh and tpi gene loci, and three assemblages (A, B and E) were identified (Table 2). Among them, 17 samples were identified as belonging to assemblage E, and these were distributed among the three provinces. Both assemblages B and E were detected in pigs from Zhejiang Province, and assemblage A and assemblage E were detected in pigs from Yunnan Province (Figure 1). Two assemblages—B together with A—have also been detected in humans and other mammals [12,14]. These results demonstrated that the pigs in Zhejiang and Guangdong provinces have the zoonotic potential to spread giardiasis to humans. Moreover, only assemblage E was detected in pigs from Guangdong Province (Figure 1). Further studies should be conducted to collect larger samples of pigs from this province to explore this topic. Assemblage E was the predominant genotype of *G. duodenalis* in pigs in the present study, which is the same as that observed in pigs in Australia and Nigeria, as well as the Shaanxi, Xinjiang and Henan provinces, in China [19,20,21,23,33]. Additionally, 13, six, and two samples were successfully amplified at the bg, gdh and tpi gene loci, respectively (Table 2 and Table 3). Only two samples were amplified at the tpi gene locus, showing the low efficiency of amplification in this study. Furthermore, seven subtypes (E1–E5 at bg, E4 at gdh, and E4 at tpi) were identified in assemblage E, and one subtype (B1 at tpi) in assemblage B (Table 2 and Table 3), but no mixed infection was detected in the present study by means of sequence analysis. These findings reveal the genetic diversity of *G. duodenalis* assemblage E in pigs, which provide basic data for further genetic research of *G. duodenalis*. Due to the fact that the feces of pigs were not treated harmlessly in our investigated area, the feces might pollute the water and the surrounding environment. People who come into contact with feces containing *Giardia* cysts increase the risk of *G. duodenalis* infection, so the facilities of farms should be designed to limit or prevent this type of exposure, especially to individuals who might be at high risk of infection. 

## 3. Materials and Methods

### 3.1. Sample Collection

In 2016, 396 fecal samples were randomly collected from pigs in seven farms in three provinces, including 124 in Zhejiang province, 72 in Guangdong province and 200 in Yunnan province. All the samples of these pigs were randomly collected from the specific farms in each province. Each fresh fecal sample was collected with a separate sterile glove, marked with the relevant numbers, gender, age and geographical origin, and then placed into the boxes filled with ice. The feces were immediately transported to the laboratory and stored at −20 °C for further analysis.

### 3.2. Genomic DNA Extraction and PCR Amplification

Each stool sample was washed with distilled water and filtered the residue was filtered with a sieve. Then, it was centrifuged at 3000 rpm for 3 min to discard the distilled water. The sediment (200 mg) was used for extracting DNA using an EZNAR Stool DNA kit (OMEGA, Biotek Inc., Norcross city, GA, USA) following the manufacturer’s instructions. Then, the DNA was stored at −20 °C before PCR amplification. All DNA samples were screened for *G. duodenalis* using nested PCR targeting the bg, gdh and tpi genes. The primers for amplification at the bg locus were G7F (5′-AAGCCCGACGACCTCACCCGCAGTGC-3′), G759-R (5′-GAGGCCGCCCTGGATCTTCGAGACGAC-3′), G99-F (5′-GAACGAACGAGATCGAGGTCCG-3′) and G609-R (5′-CTCGACGAGCTTCGTGTT-3′) [34]. For other gene loci (gdh and tpi), the primers used were GDHeF (5′-TCAACGTYAAYCGYGGYTTCCGT-3′), GDHeR (5′-GTTRTCCTTGCACATCTCC-3′), GDHiF (5′-CAGTACACCTCYGCTCTCGG-3′) and GDHiR (5′-GTTRTCCTTGCACATCTCC-3′) for the gdh gene locus [34] and AL3543 (5′-AAATIATGCCTGCTCGTCG-3′), AL3546 (5′-CAAACCTTITCCGCAAACC-3′), AL3544 (5′-CCCTTCATCGGIGGTAACTT-3′) and AL3545 (5′-GTGGCCACCACICCCGTGCC-3′) for the tpi gene locus [35]. The PCR conditions corresponded to those described in a previous report [19], and positive and negative controls were included in each amplification reaction. The PCR products were examined with 1.5% agarose gel containing GoldView^TM^ (Solarbio., Beijing city, CHN) and were observed under UV light.

### 3.3. Sequencing and Sequence Analysis

All positive PCR products were sequenced directly by the Genscript Company (Nanjing, China) and the sequence platform was used for Sanger sequencing. The obtained sequences were proofread by sight using a chromatogram and were aligned with previously reported reference sequences available in GenBank, using the Basic Local Alignment Search Tool (BLASTn) (https://blast.ncbi.nlm.nih.gov/Blast.cgi accessed on 27 August 2019) and Clustal X 1.83 to identify the assemblages of *G. duodenalis*.

### 3.4. Statistical Analysis

SPSS 19.0 (for Windows, Version, IBM Armonk Corp., New York, NY, USA) was used to analyze all the data relating to *G. duodenalis* infections. In multivariable regression analysis, each factor was included as an independent variable in the binary Logit model. The χ^2^ test was used to compare the infection rate of *G. duodenalis* in different regions, ages and breeds. All tests were two-sided, and a probability (*p*) value < 0.05 was considered statistically significant. Furthermore, 95% confidence intervals (95% CIs) were calculated to explore the correlation strength between *G. duodenalis* infections and test conditions.

### 3.5. Nucleotide Sequence Accession Numbers

The nucleotide sequences of *G. duodenalis* subtypes obtained in this study were deposited under the following accession numbers: MN434084–MN434088 for assemblage E (E1, *n* = 6; E2, *n* = 1; E3, *n* = 1 E4, *n* = 1; E5, *n* = 1) at the bg gene locus; MN434090 for assemblage E (E4, *n* = 1) at the gdh gene locus; MN434089 for assemblage E (E4, *n* = 1) and MN434091 for assemblage B (B1, *n* = 1) at the tpi gene locus. Other known *G. duodenalis* assemblages were 100% similar to previous sequences: KT922255 for assemblage A (*n* = 3); KY633473 (*n* = 3), and KJ668145 (*n* = 2) for assemblage E.

## 4. Conclusions

The present study revealed the prevalence and assemblages of *G. duodenalis* in farmed pigs in the Zhejiang, Guangdong, and Yunnan provinces in southern China, with a total prevalence of 5.3%. Moreover, region, gender and age are risk factors for *G. duodenalis* infection, which prompted us to pay more attention to these factors. Furthermore, three genotypes (assemblages A, B and E) were identified in this study, and all of them were reported as potential zoonotic genotypes. Further studies should be undertaken to collect more samples to assess the risk of animal giardiasis to human beings.

## Figures and Tables

**Table 1:** Factors associated with prevalence of *Giardia* *duodenalis* in farmed pigs.

Factor	Category	No. of Tested	No. Positive	No. of Positive (%) (95% CI)	*p*-Value
Region	Yunnan	200	5	2.5 (0.34–4.66)	0.01
	Zhejiang	124	13	10.5 (5.09–15.87)	
	Guangdong	72	3	4.2 (0–8.79)	
Gender	Female	217	6	2.8 (0.58–4.94)	0.02
	Male	179	15	8.4 (4.32–12.44)	
Age	1–3 months	87	2	2.3 (0–5.45)	0.01
	4–6 months	73	9	12.3 (4.79–19.87)	
	>6 months	236	10	4.2 (1.67–6.81)	
Total		396	21	5.3 (3.09–7.51)	

**Table 2:** Intra-assemblage substitutions in bg, gdh and tpi sequences for *Giardia duodenalis* assemblage E.

Locus	Subtype (Number)	Nucleotide Position								GenBank
bg		243	404							
	Ref. E	C	A							KY633473
	E (3)	C	A							
	E1 (6)	T	A							MN434084
	E2 (1)	T	G							MN434085
		67	153	156	160	290	293	468	495	
	Ref. E	C	T	C	T	T	C	C	C	KY633473
	E3 (1)	G	G	G	C	C	G	T	T	MN434086
	E4 (1)	G	G	G	C	T	G	T	T	MN434087
	E5 (1)	G	T	G	C	T	G	T	T	MN434088
		159	223							
gdh	Ref. E	A	C							KJ668145
	E (2)	A	C							
	E4 (1)	G	T							MN434090
	Ref. A	189	280	310						KT922255
	A (3)	A	T	G						
		10	172							
tpi	Ref. E	G	G							KJ668134
	E4 (1)	C	A							MN434089

**Table 3:** Intra-assemblage substitutions in tpi sequences for *Giardia duodenalis* assemblage B.

Locus	Subtype (Number)	Nucleotide Position	GenBank
tpi		50	
	Ref. B	A	KU892521
	B1 (1)	T	MN434091

## Data Availability

All of the obtained representative *G. duodenalis* sequences at bg, gdh and tpi loci were deposited in GenBank (https://www.ncbi.nlm.nih.gov/GenBank/accessed on 1 September 2019).
